# Lectures on Pathological Anatomy

**Published:** 1860-07

**Authors:** 


ART. VI.—*Lectures on Pathological Anatomy, delivered at Guy's Hospital during the Summer Sessions of* 1857 and 1858. By SAMUEL WILKS, M.D., F.R.C.P., Assistant Physician to Guy's Hospital, Lecturer on Pathology, and Curator of the Museum, etc., etc. 8vo., pp. 472. London, 1859.

THE work of Dr. Wilks, the latest production on pathological anatomy in the English language, has received a singular mixture of praise and condemnation at the hands of the critics. It has been warmly lauded for many obvious excellencies, and sternly rebuked for many obvious deficiencies and shortcomings. Where there is so much smoke, there must be some fire. We are free to confess that an attentive perusal of the work has impressed us with the conviction that this criticism is, in the main, well merited. As a treatise on morbid anatomy, at once full and satisfactory, it is "nowhere," having neither the advantages of a manual nor any of the broad features of a well-digested system. It is in every respect far inferior to the work of Jones and Seiveking and to the Elements of Gross, so well known in this country. We look in it in vain for those general principles the study of which is so necessary to a thorough comprehension of the subject in all rudimentary treatises. Dr. Wilks has entirely neglected everything of the kind, the very first chapter of the book opening with an account of the diseases and injuries of the bones. Such an arrangement may do very well for the advanced student, but is altogether unadapted to the wants of the young inquirer, whose progress is constantly impeded for the want of the requisite light in regard to the elementary facts of the science. Dr. Wilks has been called a "Guy's man," and it would really seem as if he had published his work exclusively for the benefit of the young men who resort to that celebrated institution, and not for the profession at large. However this may be, it is unquestionable that the frequent references to the morbid specimens and drawings in Guy's Hospital can be of no use whatever to any one; on the contrary, they only serve to encumber and disfigure the work. The lecture style of such a work is also objectionable, unless, as in the case of Watson's Practice of Medicine, it is eminently classical, animated, and entertaining, which the present production is certainly not.

Dr. Wilks runs over many of his subjects with a kind of railroad speed, hardly stopping long enough to take in anything but a piece of dry wood to keep up the steam. Whether this has arisen from a want of knowledge, an apprehension of being considered tedious or overlearned, or from a want of illustrative material in Guy's Hospital, we leave to the author himself to inform us. The fact is unmistakably as we state it.

Notwithstanding that we have found fault, and very great fault, with

Dr. Wilks's work, we can, nevertheless, heartily indorse its general character, the soundness of its doctrines, and the accuracy of his descriptions. While we cannot recommend it as a text-book, on account of the absence of rudimentary principles, so essential in a work of this kind, we are sure that no practitioner can rise from its perusal without benefit and instruction. One decided merit which the work possesses is its originality. Should another edition be called for, we hope that the author may enlarge it, and thus augment its general usefulness. British medicine certainly demands a more elaborate and comprehensive treatise on that subject; let Guy's be dropped, and a more cosmopolitan spirit be manifested.

